# Abnormal cleavage patterns in equine in vitro‐produced embryos lead to higher early pregnancy loss

**DOI:** 10.1111/evj.70004

**Published:** 2025-07-31

**Authors:** Soledad Martin‐Pelaez, Alejandro de la Fuente, Kazuki Takahashi, Hugo Monteiro, Mauricio Mendes, Stuart Meyers, Pouya Dini

**Affiliations:** ^1^ Department of Population Health and Reproduction School of Veterinary Medicine, University of California, Davis Davis California USA; ^2^ Burns Ranch Menifee California USA; ^3^ Department of Anatomy, Physiology, & Cell Biology School of Veterinary Medicine, University of California, Davis Davis California USA

**Keywords:** first mitosis, horse, IVP, pregnancy rate, time‐lapse

## Abstract

**Background:**

Despite significant advances, in vitro production (IVP) of equine embryos continues to lack standardised embryo classification criteria and is associated with increased rates of early pregnancy loss compared with in vivo‐derived blastocysts.

**Objectives:**

To evaluate morphokinetic characteristics of the first mitotic division and early embryonic development in IVP blastocysts and their association with embryo development, as well as pregnancy rate and early pregnancy loss following embryo transfer.

**Study Design:**

Retrospective analysis of archived material and clinical records.

**Methods:**

We retrospectively analysed morphokinetic characteristics of transferred IVP embryos with known pregnancy outcomes as well as those from arrested embryos. We analysed time‐lapse images of 70 transferred embryos and 114 arrested embryos to identify and compare the frequency of abnormalities during the first mitotic division (cleavage patterns) and recorded the time to vitrification or arrest. A logistic regression model with a logit link was used to evaluate pregnancy success at 14 days and early pregnancy loss (EPL) at 25 and 42 days in relation to the recorded morphokinetic characteristics in SAS.

**Results:**

Earlier vitrification (which corresponds to the time to blastocyst formation) increased the odds of pregnancy at 14 days and decreased pregnancy loss until 25 days, but not between 25 and 42 days. Abnormal cleavage patterns decreased the odds of pregnancy and increased the odds of EPL, with embryos exhibiting abnormal cleavage patterns showing a 53.3% total EPL rate compared with 22.6% for normal cleavage patterns (*p* < 0.05).

**Main Limitations:**

While our analysis provides sufficient statistical power to draw conclusions regarding the main objectives, increasing the number of embryo transfers could reveal additional interactions among morphokinetic characteristics that influence pregnancy success.

**Conclusions:**

Overall, we demonstrated a relationship between cleavage patterns during the first mitotic division, time to blastocyst formation, pregnancy rate and early pregnancy loss risk for equine IVP embryos. These features can serve as a classification method to identify and select embryos with higher pregnancy potential for transfer, contributing to reducing the gap in pregnancy rates between in vivo and in vitro‐produced embryos.

## Résumé

**Contexte:**

Malgré des avancées significatives, la production in vitro (IVP) d'embryons équins manque toujours de critères de classification embryonnaire standardisés et demeure associée à des taux élevés de perte de gestation précoce comparativement aux blastocystes produits in vivo.

**Objectifs:**

Évaluer les caractéristiques morpho‐cinétiques de la première division mitotique et du développement embryonnaire précoce de blastocystes IVP de même que leur association au développement embryonnaire, taux de gestation et de perte de gestation précoce suivant un transfert d'embryon.

**Type d'étude:**

Analyses rétrospective de matériel archivé et de dossiers médicaux.

**Méthodes:**

Les caractéristiques morpho‐cinétiques d'embryons IVP transférés avec résultats de gestation connus ont été analysées de même que celles provenant d'embryons arrêtés. Des images espacées de 70 embryons transférés et 114 embryons arrêtés ont été analysées afin d'identifier et comparer la fréquence d'anomalies durant la première division mitotique (patrons de clivage) et le temps de vitrification ou d'arrêt a été enregistré. Un modèle de régression logistique avec un lien logit a été utilisé pour évaluer le succès de gestation à 14 jours et de perte de gestation précoce à 25 et 42 jours par rapport aux caractéristiques morpho‐cinétiques enregistrés dans SAS.

**Résultats:**

Une vitrification précoce (qui correspond au temps de formation du blastocyste) augmente les chances de gestation à 14 jours et diminue la perte de gestation jusqu'à jour 25, ce qui n'est pas le cas entre jours 25 et 42. Les patrons de clivage anormaux diminuent les chances de gestation et augmentent les chances de perte de gestation précoce. Les embryons avec patrons de clivage anormaux montraient un taux total de perte de gestation précoce de 53.3.% comparativement à 22.6% pour les patrons de clivage normaux (*P* < 0.05).

**Limitations principales:**

Alors que notre analyse possède suffisamment de pouvoir statistique pour tirer des conclusions par rapport aux objectifs primaires, un nombre supérieur de transferts d'embryons pourrait révéler des interactions additionnelles en termes de caractéristiques morpho‐cinétiques influençant les succès de gestation.

**Conclusions:**

Somme toute, nous avons démontré une relation entre les patrons de clivage durant la première division mitotique, le temps de formation du blastocyste, le taux de gestation et le risque de perte de gestation précoce pour des embryons IVP équins. Ces caractéristiques peuvent servir comme méthode de classification afin d'identifier et sélectionner des embryons ayant un potentiel de gestation supérieur pour un transfert, contribuant à réduire la marge de taux de gestation entre les embryons produits in vivo et in vitro.

## 1 INTRODUCTION

Assisted reproductive techniques in horses have gained popularity in recent years, especially the use of transvaginal aspiration of oocytes (TVA) for oocyte retrieval and subsequent in vitro embryo production (IVP) via Intracytoplasmic Sperm Injection (ICSI).^1^ The improvement of in vitro oocyte maturation and culture systems has led to an increase in the number of blastocysts produced per TVA/ICSI session, with the latest reports reaching two embryos per TVA procedure.^2^, ^3^ Despite the increasing success of equine in vitro embryo production, 14‐day pregnancy rates for IVP embryos are reported to be around 77.7%, with a pregnancy loss rate of 15%–20%,^3^, ^4^ compared with in vivo derived embryo (IVD) rates of 85% for pregnancy and 11% for early embryonic loss.^5^ The differences in pregnancy potential are most likely attributable to embryo quality. Therefore, establishing a grading system for IVP embryo quality—similar to those used for IVD embryos—could facilitate the selection of the most suitable embryos for transfer and serve as a valuable tool to improve IVP pregnancy rates.

One of the challenges encountered in IVP is the different morphological and developmental features these embryos present compared with IVD embryos. For example, while culturing in vitro, IVP embryos fail to produce the glycoprotein capsule observed around their in vivo counterparts, and they also present a higher percentage of apoptotic cells.^6^, ^7^, ^8^ It has also been shown that day seven IVP embryos were more compact, lacked a blastocele cavity, had lower cell numbers, decreased inner cell mass/trophectoderm differentiation, and more intermixed epiblast and primitive endoderm cells compared with IVD embryos.^8^ The relationship between embryo grades and pregnancy rate after transfer has been well studied and documented for IVD embryos, not only in horses^9^, ^10^, ^11^, ^12^ but also in cattle,^13^, ^14^ with only the highest quality embryos yielding acceptable pregnancy rates. However, marked morphological and developmental differences of equine IVP compared with IVD embryos impede the use of guidelines available for the classification of IVD embryos.^9^, ^15^ Nevertheless, IVP offers the opportunity to observe early embryonic development from the first mitotic division to the blastocyst stage, whereas IVD embryos can only be collected after entering the uterine lumen after 6 days post fertilisation, leaving the earlier developmental stages essentially unknown in clinical settings.^16^ In this regard, the use of time‐lapse (TL) imaging incubators has a clear advantage over conventional static timepoint observations, as it allows observation of every event of embryo development almost in real time without disrupting the culture conditions.^17^ TL has provided valuable insights into the events leading to blastocyst formation in the horse, showing an association between the time of cytoplasmic extrusion, time to first cleavage, and 4‐cell formation with the likelihood of zygotes becoming blastocysts and presence of embryo pulsing as an indicator of the blastocyst stage.^15^, ^18^, ^19^, ^20^, ^21^, ^22^

These findings in the horse are consistent with those in other species, where embryo grading based on embryonic morphokinetics is more common.^23^, ^24^, ^25^ For example, in humans, the degree of morula compaction has been linked to the likelihood of advancing to the blastocyst stage, as well as the incidence of mosaicism.^26^ Other studies have also associated the direct cleavage pattern (the division of a zygote into three blastomeres) and the indirect cleavage pattern (the division into two cells and subsequent fusion into one cell again) with lower implantation potential in humans^27^, ^28^ and cattle.^29^ In the horse, direct cleavage has been associated with the exposure to di‐(2‐ethylhexyl) phthalate (DEHP) during in vitro maturation,^30^ although it has also been observed, to a lesser extent, in untreated oocytes that yielded both arrested embryos and blastocysts.^18^, ^19^, ^21^ These studies highlight the importance of the first mitotic division in proper embryonic development. The characteristics of this cell division have been linked to pregnancy potential in other species^29^, ^31^, ^32^, ^33^, ^34^, ^35^, ^36^ but not in horses, where only one study reported the transfer of two embryos with abnormal cleavage patterns, yielding one pregnancy.^19^ Therefore, in this study, we aimed to evaluate the association of morphokinetic characteristics of the first mitotic division and early embryonic development in IVP blastocysts with pregnancy rate and early pregnancy loss after embryo transfer. The future outlook of the study is to lay the groundwork for establishing classification criteria for IVP embryos.

## 2 MATERIALS AND METHODS

### 2.1 Oocyte collection

Oocytes were obtained for commercial embryo production purposes by two referring clinics via routine transvaginal aspiration of follicles from 38 mares in 49 different cycles, and sent to the laboratory for IVP. All oocytes were part of the clinical programme of the Veterinary Assisted Reproduction Laboratory of the University of California, Davis, and no research animals were used. EquiPlus OPU Recovery Medium (Minitube) at 37°C was used for equine oocyte aspiration, and cumulus‐oocyte complexes (COCs) searching was done at room temperature. The oocytes were transported overnight to the laboratory in a temperature‐controlled container (MicroQ, Micro Q Technologies) at 22°C. All oocytes were held in commercial holding media (EquiHold, Minitube USA Inc.) for ~24 h before being transferred to maturation media.^37^

### 2.2 Oocyte maturation, ICSI and culture

After the holding period, our routine equine IVP protocols were followed, as previously described.^38^, ^39^ Briefly, groups of two COCs were transferred to the maturation medium in 25 μL droplets under oil overlay (Fujifilm Irvine Scientific) and matured for 28–32 h at 38.2°C in a humidified atmosphere of 5.8% CO_2_, 5% O_2_ and 89.2% N_2_. The maturation medium consisted of 34% Global® (Cooper Surgical Inc), 50% DMEM/F‐12 (Thermo Fisher Scientific), 25 μg/mL gentamicin (Thermo Fisher Scientific), 0.1 mM sodium pyruvate (Sigma‐Aldrich), 6% fetal bovine serum (FBS, R&D Systems), 10 μL/mL insulin‐transferrin‐selenium solution (Thermo Fisher Scientific), 10% dominant follicle follicular fluid, 8.8 mU/mL ovine FSH (National Hormone And Peptide Program) and 1.1 mU/mL porcine somatotropin (Harbor‐UCLA Research and Education Institute). Dominant follicular fluid was aspirated from the pre‐ovulatory follicles of mares. In brief, mares were monitored, and once a dominant follicle reached a diameter greater than 35 mm, an ovulation induction dose of deslorelin acetate (SucroMate™ Equine, Dechra) was administered. Twenty‐four hours later, the follicular fluid was aspirated, filtered using a 0.22 μm filter and stored at −80°C until the preparation of the maturation medium. The overall maturation rate was 64%.

After maturation, COCs were stripped of cumulus cells by repeated pipetting in 0.02 g/mL hyaluronidase in G‐MOPS (Vitrolife) supplemented with 10% FBS, and maturation status was assessed by evaluation of the presence of a polar body. Only matured oocytes were injected with frozen–thawed sperm from 26 different stallions selected by the clients. Sperm selection was done using the swim‐up method.^38^, ^40^ Following intracytoplasmic sperm injection (ICSI) using conventional needles (Cook Medical LLC), the injected oocytes were cultured individually within a 25 μL droplet with an oil overlay in MIRI®TL time‐lapse imaging incubators (Esco Technologies) at 38.2°C under 5.8% CO_2_, 5% O_2_ and 89.2% N_2_. The culture medium consisted of 54% DMEM/F‐12, 40% Global® (Cooper Surgical Inc), 6% FBS, 10 μL/mL insulin‐transferrin‐selenium solution and 0.1 mM sodium pyruvate.

On day four post‐ICSI, 75% of the culture medium was replaced, and the embryos were cultured until the blastocyst stage (day 7–10), identified by the formation of a clear trophectoderm layer and visible pulsing.^22^ The overall blastocyst formation rate (blastocyst/injected oocytes) was 31%. All blastocysts were then vitrified as previously described.^38^ Briefly, a two‐step vitrification protocol was used, submerging the embryo for 5 min in solution A (DMEM F12‐ Hepes, 1.5 M ethylene glycol, 20% FBS) followed by 40 s in solution B (DMEM F12—Hepes, 7.0 M ethylene glycol (Sigma‐Aldrich) with 0.6 M galactose (Sigma‐Aldrich), 20% FBS (R&D System)). Embryos were then placed in Cryolocks (Biotech INC) and plunged into liquid nitrogen.

### 2.3 Time‐lapse imaging evaluation

**FIGURE 1:**
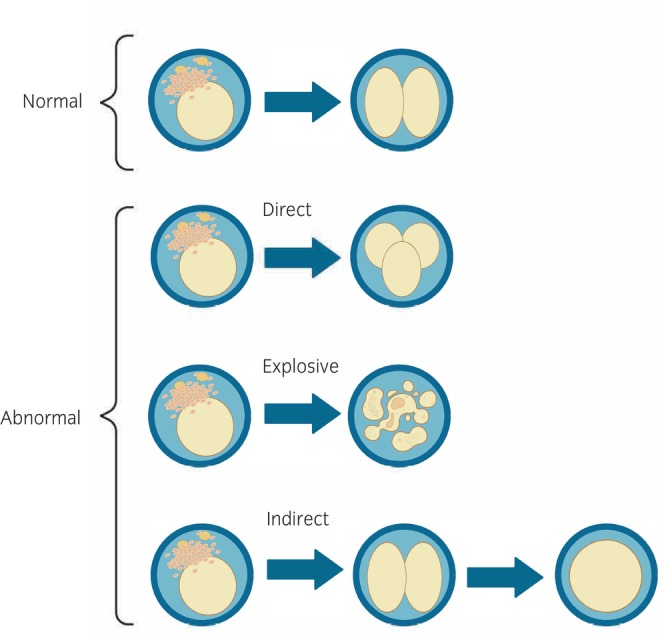
Types of cleavage pattern in the first mitotic division. Polar bodies and cytoplasmic extrusion are shown in all zygotes as a marker of normal equine maturation and early embryonic development.

Embryo selection was based on the availability of pregnancy outcomes evaluated by the clinicians in embryos produced and transferred between 2022 and 2024. TL imaging was performed using the MIRI®TL incubators, and recordings were evaluated by a single blinded reviewer. Time from injection to vitrification (time to blastocyst formation) was recorded. The cleavage pattern of the first division was classified as normal (two blastomeres formed; Figure 1 and Video S1) or abnormal (direct cleavage, indirect cleavage or explosive cleavage; Figure 1 and Video S2).^28^, ^29^ Direct cleavage was characterised by the formation of three blastomeres at the first division, indirect cleavage involved the formation of two daughter blastomeres that fused before resuming normal mitotic division, and explosive cleavage occurred when multiple blastomeres of equal or unequal sizes appeared during the first mitotic division (Figure 1).

To evaluate the overall rate of normal and abnormal cleavage and the influence of cleavage patterns in embryonic development, all the injected oocytes that did not develop into blastocysts in the same TVA/ICSI session were also retrospectively analysed and categorised as arrested embryos. The cleavage patterns of all zygotes that underwent cleavage were analysed, along with the timing of arrest. The arrest time was classified as early (up to the 8‐cell stage, during embryonic genome activation^41^), mid (from the 16‐cell stage to morula) or late (after morula compaction).

### 2.4 Embryo transfer

All embryos were thawed (single‐step) in the equine commercial holding medium (EquiHold, Minitube USA Inc.) at 37°C for 5 min prior to loading in 0.25 mL sterile straws and transferred into suitable recipient mares on day four post‐ovulation, following a thorough evaluation of the recipients. A recipient was considered suitable when the preceding oestrus cycle exhibited at least 3 days of high uterine oedema (>2), and on the day of embryo transfer, there was no evidence of fluid, air or high oedema (>1) in the uterus with the presence of a corpus luteum.^5^ All embryos were frozen by the same embryologist, thawed by two experienced embryologists, and transferred by equine reproduction specialists with extensive experience in embryo transfer procedures (>10 years) with the Wilsher‐Allen technique.^42^

### 2.5 Pregnancy rate and early pregnancy loss

The available breeding records for the transferred embryos were reviewed. The pregnancy rate was defined as the total number of recipients diagnosed as pregnant at 14 days of embryonic age divided by the total number of embryo transfers. Early pregnancy loss (EPL) was described as the number of nonpregnant recipients at 25 and 42 days divided by the number of pregnant recipients at the previous exam.

### 2.6 Data analysis

The percentages of zygotes with normal and abnormal cleavage were compared between those that developed into blastocysts and those that were arrested using a chi‐square test. Additionally, the percentages of embryos with normal and abnormal cleavage, resulting in pregnant or nonpregnant recipients, were compared across three time points (14, 25 and 42 days). A test for independence was performed to check the association of time to vitrification, cleavage pattern, stallion and mare with pregnancy rate at days 14, 25 and 42 using a chi‐square test in PROC FREQ of SAS 9.4 (SAS Institute Inc.). The association of all variables synergistically to pregnancy outcomes, such as time to vitrification, cleavage pattern, stallion and mare, was subsequently tested in a logistic regression model with stepwise model selection based on the lowest Akaike Information Criterion corrected for sample size (AICC) in PROC LOGISTIC of SAS. The model was as follows:

$$\text{Logit}\;\left(P\;\left(\text{Pregnancy}=\mathrm{Yes}\right)\right)=\mu +{\text{Time to Vitrification}}_{i}+{\text{Cleavage Pattern}}_{j}+{\text{Stallion}}_{k}+{\text{Mare}}_{l}+\varepsilon_{\text{ijkl}},$$

where $\mu \;\text{was the overall mean}\;\log\;\text{odds of detection}\;\left(\text{intercept}\right)$, followed by ‘Time to Vitrification’ (*i* = 7 to 10), ‘Cleavage Pattern’ (*j* = Normal or Abnormal), ‘Stallion’ (*k* = 1–22), ‘Mare’ (*l* = 1–34) and a random error. The final model for the prediction of pregnancy at days 14, 25 and 42 was also fitted in a generalised linear mixed model using the PROC GLIMMIX in SAS to estimate the probabilities of pregnancy based on cleavage pattern. The ‘ilink’ option in SAS was used to calculate probabilities from log (odds) for specific discussions within the scope of the study only. The standard error of the mean (SEM) for the probabilities were obtained by applying the delta method in SAS on the log(odds) SEM. Furthermore, pregnancy loss rates were calculated as the complement of pregnancy success at each timepoint (i.e., Loss Rate = 1 − Success Rate). To assess the relative risk of pregnancy loss associated with abnormal cleavage patterns compared with normal cleavage patterns, we calculated it at days 14, 25 and 42. The relative risk was defined as the ratio of pregnancy loss rates for embryos with abnormal cleavage to those with normal cleavage. These calculations allowed us to quantify the magnitude of increased pregnancy loss associated with abnormal cleavage patterns on different days. Statistical significance was considered when *p* ≤ 0.05.

## 3 RESULTS

### 3.1 Time‐lapse evaluation

#### 3.1.1 Blastocyst

A total of 70 in vitro‐produced embryos that reached the blastocyst stage were evaluated. Abnormal cleavage patterns were observed in 41% (29/70) of all embryos. Of these, 21% (6/29) exhibited direct cleavage into three or four blastomeres, 3% (1/29) displayed indirect cleavage and 48% (14/29) presented explosive cleavage. The remaining embryos exhibited either a combination of direct and indirect cleavage (17%; 5/29) or a combination of explosive and indirect cleavage (10%; 3/29).

#### 3.1.2 Arrested embryos

A total of 114 arrested embryos from the same cycles were evaluated. The overall incidence of abnormal cleavage was 40% (47/114) in arrested embryos, which was not statistically different from the incidence of abnormal cleavage in the blastocysts (*p* = 0.9). There was no association between the cleavage abnormality and the time of embryonic arrest (*p* = 0.9). The stage of arrest was not different between arrested embryos with normal and abnormal cleavage patterns (early = 52% and 54%, *p* = 0.8; mid = 15% and 16%, *p* = 0.8; late = 33% and 29%, *p* = 0.5; for abnormal and normal cleavage, respectively).

### 3.2 Pregnancy success and early pregnancy loss

Information regarding pregnancy status at 14 days was available for all the 70 embryos transferred, with a total of 52 (74%) mares confirmed pregnant. At the 25‐day exam, data was available for 50 of the 52 initial pregnancies, with 82% of mares still pregnant (41/50). At 42 days, pregnancy records were available for 37 of the 41 pregnancies, with 83.8% of mares remaining pregnant (31/37). Overall, EPL, defined as confirmed pregnancies lost by either 25 or 42 days, was 32%. Cleavage pattern was associated with EPL rate (*p* = 0.04), where abnormal cleavage patterns resulted in 53.3% EPL (8/15) whereas normal cleavage patterns yielded 22.6% EPL (7/31).

### 3.3 Relationship between cleavage patterns, time to vitrification and pregnancy outcomes

**FIGURE 2:**
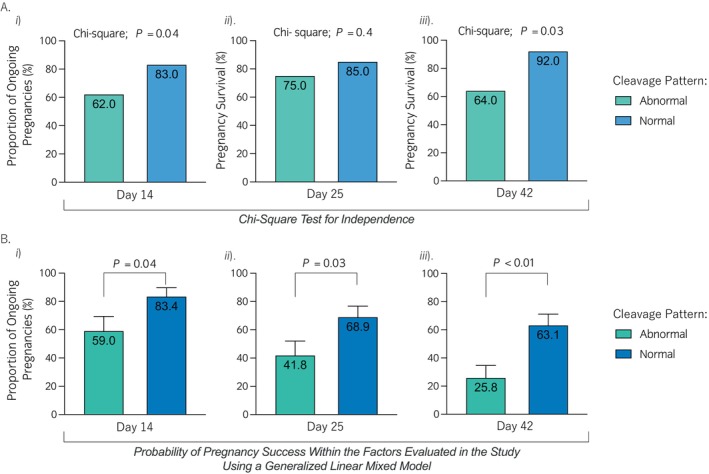
(A) Pregnancy rate at 14 (i), and pregnancy survival at 25 (ii) and 42 (iii) days for embryos with normal and abnormal cleavage patterns. The pregnancy rate was calculated as the number of pregnant recipients divided by the total number of embryos transferred with or without cleavage abnormalities, respectively. Pregnancy survival was calculated as the percentage of pregnancies surviving from the pregnancies present at the previous stage. Rates were compared between embryos with and without abnormal cleavage. (B) For illustration within the scope of this study only, probabilities were estimated from the inverse link function of the logit link in PROC GLIMMIX in SAS for the probability of ongoing pregnancy at days 14, 25 and 42 based on abnormal versus normal cleavage patterns. Bars represent the group means and whiskers the standard error of the mean derived from the delta method on the log(odds) standard errors. Statistical differences were considered when *p* ≤ 0.05. (i) Probability of pregnancy at day 14 based on abnormal versus normal cleavage patterns; (ii) Probability of ongoing pregnancy at day 25 based on abnormal versus normal cleavage patterns; and (iii) Probability of ongoing pregnancy at day 42 based on abnormal vs. normal cleavage patterns.

Test for independence showed that only time to vitrification and cleavage pattern were associated with pregnancy outcomes. Recorded pregnancy rates between embryos with normal and abnormal cleavage are presented in Figure 2A. Subsequently, the contribution of each variable to pregnancy outcomes was tested synergistically in the full model. Each additional day to vitrification decreased the odds of pregnancy at both 14 and 25 days of gestation but not at 42 days (Tables 1 and 2). The odds of pregnancy decreased by 51.6% for each additional day to vitrification for pregnancy at day 14, and by 45.6% for each additional day to vitrification for pregnancy at day 25. Cleavage pattern had a significant effect on pregnancy outcomes across all the examined days (*p =* 0.04, *p =* 0.03 and *p* < 0.01 for 14, 25 and 42 days, respectively), highlighting its importance relative to the other evaluated factors (Table 2). The translation of these odds to the probability of pregnancy in the scenario of the factors evaluated in the study is presented in Figure 2B. At day 14, embryos with abnormal cleavage had lower odds of pregnancy success when compared with those with normal cleavage patterns (probability of pregnancy = 59 vs. 83%, OR = 0.29, 95% CI = 0.09–0.98, *p* = 0.04), with a calculated relative risk of not establishing pregnancy 2.33 times higher for abnormal versus normal cleavage pattern. At day 25, embryos with abnormal cleavage also had lower odds of pregnancy success when compared with those with normal cleavage patterns (probability of pregnancy = 42 vs. 69%, OR = 0.32, 95% CI = 0.11–0.97, *p* = 0.03), with a relative risk of not being pregnant 1.87 times higher (87% increase) for abnormal versus normal cleavage pattern. At day 42, during which only the cleavage pattern was significant to the final model, embryos with abnormal cleavage had even lower odds of pregnancy success when compared with those with normal cleavage patterns (probability of pregnancy = 26 vs. 63%, OR = 0.22, 95% CI = 0.07–0.64, *p* < 0.01). Embryos with abnormal cleavage in this final check day had 1.97 times the relative risk of not being pregnant compared with those with normal cleavage embryos.

**TABLE 1:** Number of embryos by vitrification day, type of cleavage and subsequent pregnancy outcome.

Day of vitrification	Cleavage patterns	Total embryos vitrified	Pregnancies at day 14	Ongoing pregnancies at day 25	Ongoing pregnancies at day 42
Day 7	Normal cleavage	9	9/9	9/9	7/7
Day 8		21	16/21	12/16	10/12
Day 9		5	5/5	4/5	4/4
Day 10		6	4/6	4/4	3/3
Day 7	Abnormal cleavage	7	7/7	6/6	5/6
Day 8		13	8/13	4/7	1/3
Day 9		5	2/5	1/2	0/1
Day 10		4	1/4	1/1	1/1

**TABLE 2:** Final model with factors deemed critical for the odds of pregnancy at days 14, 25 and 42 after a logistic regression in SAS with stepwise variable selection based on the lowest AICC.

	Logistic regression with stepwise variable selection					
				95% CI		
Parameters^a^	*β* coefficient	*β* SEM	OR	Lower	Upper	*p‐*value
Pregnancy at day 14						
Time to vitrification, days	−0.73	0.29	0.48	0.28	0.85	0.01
Normal cleavage	Ref					
Abnormal cleavage	−1.25	0.62	0.29	0.09	0.98	0.04
Pregnancy at day 25						
Time to vitrification, days	−0.61	0.28	0.54	0.32	0.93	0.03
Normal cleavage	Ref					
Abnormal cleavage	−1.13	0.56	0.32	0.11	0.97	0.03
Pregnancy at day 42						
Normal cleavage	Ref					
Abnormal cleavage	−1.54	0.56	0.22	0.07	0.64	<0.01

^a^ Parameters were defined as those that remained in the final model after stepwise selection using the PROC LOGISTIC in SAS, given a *p* ≤ 0.05 and lowest Akaike Information Criterion corrected for sample size (AICC).

## 4 DISCUSSION

The expansion of assisted reproductive techniques in domestic species presents both challenges and opportunities to deepen our understanding of early embryonic development. The use of TL imaging to study the precise timing and morphology of early embryonic development has garnered significant interest across multiple species, as it has proven useful for predicting the potential of a fertilised oocyte developing into a blastocyst, its pregnancy potential and even pregnancy survival.^43^, ^44^, ^45^, ^46^, ^47^, ^48^ In the case of horses, the fundamental differences between IVP embryos and their IVD counterparts highlight the need for further study of embryo developmental characteristics. In this study, we evaluated the use of TL imaging as a new method to investigate the relationship between some of the morphokinetic parameters of the early equine embryo and its pregnancy potential, with a particular focus on the first mitotic division.

In our study, we found a relationship between the time required to reach the blastocyst stage (equivalent to the time of vitrification) and the pregnancy rate at 14 and 25 days, but not at 42 days. Several studies have noted delays in the development of IVP embryos in comparison to IVD embryos, with blastocysts produced in vitro showing altered cell lineage differentiation and morphological differences, such as the absence of a capsule and differential gene expression at equivalent developmental time points to their in vivo counterparts.^7^, ^8^, ^49^ However, these differences were mitigated when IVP embryos were transferred into the uterus.^8^, ^50^ This ‘rescue effect’ by the uterus suggests that while the in vitro culture environment supports embryo development, it may lack essential components required for full embryonic growth, such as uterocalin, which aids in capsule formation and early embryonic development during the horse's prolonged preimplantation period.^51^, ^52^ Furthermore, the developmental speed of IVP embryos has been associated with the pregnancy or foaling rate, with embryos that reach the blastocyst stage within 7 days after ICSI having better outcomes, while those taking more than 9 days have reduced pregnancy potential or foaling rates.^3^, ^53^, ^54^ This is consistent with our findings, which show that the time to vitrification significantly affects pregnancy potential at 14 and 25 days. However, we did not detect an effect of developmental speed on the pregnancy losses between 25 and 42 days of gestation. This information suggests that the speed of early embryonic development influences embryonic survival during critical timepoints for cell lineage determination and embryonic growth. Equine early embryonic development comprises two key cell lineage differentiations. The first involves the formation of trophectoderm cells, which eventually contribute to the placenta.^55^ The second differentiation involves the formation of the inner cell mass, which develops into the embryo, and the primitive endoderm, which forms the primitive yolk sac.^8^, ^50^, ^56^ In horses, the differentiation of the primitive endoderm is more scattered in IVP embryos, and full ICM compaction and differentiation only take place following embryo transfer.^8^, ^50^, ^57^ Future studies are needed to understand the molecular mechanisms underlying the effects of initial developmental speed on both early and late embryonic loss and its relationship to cell lineage determination.

Another factor we analysed in this study was the morphology of the first mitotic division, which has been extensively studied in other species. In cattle, a 47% incidence of abnormal cleavage patterns has been reported,^58^ similar to findings in rhesus macaques (50%),^59^ and consistent with our observed 41% abnormal cleavage rate. In cattle and humans, abnormally cleaved zygotes exhibit reduced developmental potential, with lower blastocyst formation rates compared with normally cleaved embryos.^58^, ^60^, ^61^, ^62^ The fertilisation methods employed in these studies included traditional in vitro fertilisation and intracytoplasmic sperm injection (ICSI), without a comprehensive analysis of the impact of the fertilisation method on the cleavage pattern—a factor that warrants further investigation, as polyspermy can yield multipolar mitosis.^62^ Interestingly, we found a similar incidence of abnormal cleavage in zygotes that developed into blastocysts as those that were arrested, suggesting that abnormal cleavage in horses does not necessarily impede embryonic genome activation at the 4–8 cell stage^41^ or the first and second cell lineage differentiation leading to the morula and blastocyst stages.^63^ This may indicate a difference in the downstream effects of abnormal cleavage patterns in horses or a more efficient embryo rescue mechanism to correct these errors. Similar observations have been made in rhesus macaques, despite differences in cattle and humans.^59^ However, we found that abnormal cleavage negatively affected pregnancy rates at 14 days, implying that while abnormal cleavage is not predictive of blastocyst formation in horses, it likely impacts early embryo competency and reduces survival post‐transfer.

In addition, pregnancy rates are reported to be lower for embryos with abnormal cleavage in both cattle and humans,^32^, ^47^, ^58^, ^62^, ^64^, ^65^ consistent with our findings. Here, we observed an association between the characteristics of the first cleavage and pregnancy potential; embryos with abnormal cleavage patterns had only a 26% probability of successful pregnancy, whereas normally cleaved embryos had a 63% chance at 42 days (1.97 times the risk of pregnancy loss [97%]). Nevertheless, several studies have documented that abnormal cleavage embryos can still establish pregnancies in humans, albeit at reduced rates.^27^, ^62^, ^64^, ^65^, ^66^ In horses, one study reported that out of two embryos with abnormal cleavage transferred, one successfully established a pregnancy.^19^ This finding aligns with our results, where seven embryos with abnormal cleavage patterns successfully established pregnancies.

Although TL imaging enables the observation of cleavage patterns, it does not elucidate the underlying aetiology. Morphologically abnormal cleavage patterns may result from mitotic anomalies such as lagging chromosomes leading to micronuclei, the formation of dual mitotic spindles, failure of pronuclear migration and syngamy or cytokinesis failure independent of chromosomal errors.^67^, ^68^, ^69^ Errors in chromosomal segregation can lead to aneuploidies, which are passed on to the entire blastomere's clone lineage.^69^ In other species, embryos with abnormal cleavage patterns present a higher degree of mosaicism and aneuploidy.^70^, ^71^, ^72^, ^73^ It is worth noting that in humans, embryos with chromosomal abnormalities can still implant and, albeit at lower rates, result in seemingly healthy offspring.^74^, ^75^ Thus, chromosomal missegregation cannot be ruled out as a potential underlying cause or consequence of abnormal cleavage, even in cases where pregnancies are established. The striking effect of abnormal cleavage patterns on pregnancy loss shown in our study suggests that the underlying anomalies may carry over to the later stages of embryonic development, leading to EPL. Aneuploidy has been linked to EPL in the horse, with over 20% of EPL cases presenting aneuploidies.^76^, ^77^ The higher incidence of pregnancy wastage in abnormal cleavage embryos in our study could be due to abnormal chromosomal copy numbers, which can lead to lethal embryonic phenotypes or cellular apoptosis. Aneuploidy alters gene dosage, causing proteotoxic stress and/or replication stress, ultimately triggering apoptosis.^63^ Such cell death results in embryonic arrest in a substantial number of cases.^78^, ^79^ However, later‐stage embryonic or fetal death may also occur, as evidenced by the high prevalence of aneuploidy in naturally occurring pregnancy losses in horses,^77^ consistent with findings in other species.^80^, ^81^ The incidence of aneuploidies and mosaicism in embryos and lost conceptuses derived from abnormally cleaved embryos remains unknown in the horse. However, the high EPL observed in our study underscores the need for further research to elucidate the mechanisms underlying abnormal cell divisions and their effects on chromosomal numbers in horses.

Overall, we demonstrated that morphokinetic parameters observed by TL imaging are a useful indicator of the pregnancy potential of equine IVP embryos. Accurate classification of IVP embryos could help narrow the gap between the pregnancy rates of IVP and IVD embryos, increase breeding profitability and optimise recipient mare usage. However, studies linking embryo grading to pregnancy outcomes remain scarce in equine reproduction. A study evaluating equine IVP embryos using micrograph‐based grading revealed high interobserver variability, with 34.2%–44.6% agreement among observers and reliable pregnancy prediction limited to the poorest morphological grades.^15^ In contrast, recent research on the morphological classification of embryos on vitrification demonstrated strong predictive value for pregnancy potential and pregnancy loss, suggesting its utility for embryo classification on the transfer day, although interobserver reliability was not assessed.^82^ Another study employed TL imaging to assess embryo quality with only 11 transferred embryos.^19^ Although this study did not aim to investigate the association between cleavage patterns and EPL, it documented the presence of abnormally cleaved embryos, corroborating our findings and highlighting the utility of TL imaging for embryo classification. Overall, morphokinetic grading and the transfer of ‘*superior quality embryos*’ could enhance the pregnancy rates of IVP embryos, aligning them more closely with those of IVD embryos. This is evident in our study, where embryos with normal cleavage achieved pregnancy rates exceeding 80%, and ongoing pregnancies remained significantly higher than those from embryos with abnormal cleavage.

In conclusion, our results suggest that embryo classification based on cleavage patterns and time to vitrification is a practical tool for making informative decisions about embryo transfer potential outcomes and the choice of the recipient. The ability to classify embryos prior to thawing enhances clinical preparation and improves the management of client expectations, providing tangible benefits for the equine breeding industry while advancing assisted reproductive technologies.

## FUNDING INFORMATION

This project was supported by the Center for Equine Health with funds provided by the State of California satellite wagering fund and contributions by private donors and the John P. Hughes Endowments.

## CONFLICT OF INTEREST STATEMENT

The authors declare no conflicts of interest.

## AUTHOR CONTRIBUTIONS

**Soledad Martin‐Pelaez:** Conceptualization; investigation; writing – original draft; visualization; validation; project administration. **Alejandro de la Fuente:** Conceptualization; investigation; methodology; writing – review and editing; data curation; validation. **Kazuki Takahashi:** Conceptualization; investigation; writing – review and editing; methodology; validation. **Hugo Monteiro:** Conceptualization; methodology; formal analysis; writing – review and editing; visualization; validation. **Mauricio Mendes:** Methodology; investigation. **Stuart Meyers:** Conceptualization; writing – review and editing. **Pouya Dini:** Conceptualization; supervision; resources; writing – review and editing; funding acquisition; project administration.

## DATA INTEGRITY STATEMENT

Pouya Dini had full access to all the data in the study and takes responsibility for the integrity of the data and the accuracy of data analysis.

## ETHICAL ANIMAL RESEARCH

Research ethics committee oversight not required by this journal: Archived and clinical data were used.

## INFORMED CONSENT

Explicit owner consent for inclusion of animals in this study was not stated. All owners or their agents were made aware that case material information may be used anonymously for the purposes of clinical research.

## Supporting information


**Video S1.** The cleavage pattern of the first division was classified as normal (two blastomeres formed).

**Video S2.** The cleavage pattern of the first division was classified as abnormal (direct cleavage, or explosive cleavage).

## Data Availability

The data that support the findings of this study are available upon reasonable request from the corresponding author: Open data sharing exemption granted by the editor.
